# Diagnostic Advances in Leptospirosis: A Comparative Analysis of Paraclinical Tests with a Focus on PCR

**DOI:** 10.3390/microorganisms13030667

**Published:** 2025-03-15

**Authors:** Elena Ciurariu, Catalin Prodan-Barbulescu, Diana-Maria Mateescu, Paul Tutac, Virgiliu-Bogdan Sorop, Monica Susan, Norberth-Istvan Varga

**Affiliations:** 1 Department of Functional Sciences, Physiology, Centre of Immuno-Physiology and Biotechnologies (CIFBIOTEH), “Victor Babeş” University of Medicine and Pharmacy, Eftimie Murgu Square, No. 2, 300041 Timisoara, Romania; ciurariu.elena@umft.ro; 2 Department I—Discipline of Anatomy and Embryology, Faculty of Medicine, “Victor Babeş” University of Medicine and Pharmacy Timisoara, Eftimie Murgu Square, No. 2, 300041 Timisoara, Romania; 3 Doctoral School, Department of General Medicine, “Victor Babeş” University of Medicine and Pharmacy, Eftimie Murgu Square, No. 2, 300041 Timisoara, Romania; diana.mateescu@umft.ro (D.-M.M.); norb-erth.varga@umft.ro (N.-I.V.); 4 Toxicology and Molecular Biology Department, “Pius Brinzeu” Clinical Emergency County Hospital, 300723 Timisoara, Romania; paul.tutac@gmail.com; 5 Department of Obstetrics and Gynecology, “Victor Babeş” University of Medicine and Pharmacy, Eftimie Murgu Square, No. 2, 300041 Timisoara, Romania; bogdansorop@yahoo.com; 6 Department of Internal Medicine I, Centre for Preventive Medicine, “Victor Babes” University of Medicine and Pharmacy, Eftimie Murgu Square, No. 2, 300041 Timisoara, Romania; susan.monica@umft.ro

**Keywords:** leptospirosis, *Leptospira*, leptospirosis diagnosis, PCR, MAT, Microscopic Agglutination Test, polymerase chain reaction, Weil’s disease

## Abstract

Leptospirosis is a zoonotic disease with a varied clinical presentation that can mimic other infectious diseases, posing diagnostic challenges. While the Microscopic Agglutination Test (MAT) remains the gold standard for serological diagnosis, its limitations have led to increasing interest in polymerase chain reaction (PCR) as a rapid and sensitive diagnostic tool. This systematic review evaluates the role and clinical applications of PCR for diagnosing human leptospirosis. We analyzed the sensitivity and specificity of PCR, compared its performance with other diagnostic tests, and assessed the comparative utility of blood and urine samples for PCR testing. Our findings demonstrate that PCR has a high sensitivity and specificity, particularly in the early stages of the disease. Combining PCR with serological tests like MAT can maximize the diagnostic accuracy across different stages of illness. We recommend that PCR be considered a first-line diagnostic test for suspected leptospirosis, especially when rapid diagnosis is crucial. Further research is needed to standardize PCR protocols and explore its potential in differentiating *Leptospira* species and serotypes. By leveraging the strengths of PCR and combining it with other diagnostic methods, we can significantly improve the diagnosis and management of leptospirosis.

## 1. Introduction

Leptospirosis is a globally significant zoonotic disease caused by infection with pathogenic spirochetes of the genus *Leptospira* [1,2]. These are Gram-negative, spiral-shaped bacteria that thrive in warm, moist environments [3,4,5,6,7]. The disease was first described in 1886 by the German physician Adolf Weil, who observed a severe illness characterized by fever, jaundice, and hemorrhage [8,9]. However, the causative bacterium, *Leptospira*, was only discovered and described in 1915 by Inada et al., who identified its transmission through water contaminated with animal urine, primarily from rodents [8,9]. *Leptospira* species are found worldwide in various environments, including soil and water, particularly in tropical and subtropical regions [7,10]. The bacteria are maintained in the renal tubules of reservoir animals, such as rodents, livestock, and dogs, and shed in their urine, contaminating the environment and facilitating transmission to humans [7,10,11].

Diagnosing leptospirosis presents a significant challenge due to its highly variable and non-specific clinical presentation, which mimics a wide range of other infectious diseases [12,13,14]. This often leads to delays in diagnosis and appropriate treatment, potentially resulting in severe complications and even death.

For decades, the Microscopic Agglutination Test (MAT) has been considered the gold standard for serological diagnosis of leptospirosis [14,15,16]. MAT detects antibodies against *Leptospira* in the patient’s serum by observing agglutination (clumping) of live leptospires in the presence of specific antibodies. Its long-standing use is attributed to its high specificity and ability to identify different serovars. However, MAT has limitations. It requires paired serum samples collected several weeks apart to demonstrate a rise in antibody titers, leading to delays in diagnosis. Additionally, MAT requires specialized laboratory facilities and expertise, making it less accessible in resource-limited settings where leptospirosis is most prevalent [17,18,19]. 

In recent years, the advent of molecular techniques, particularly polymerase chain reaction (PCR), has revolutionized the diagnosis of many infectious diseases. This technique amplifies specific regions of DNA, allowing for the detection and identification of even minute quantities of genetic material from a sample. PCR offers the advantage of directly detecting *Leptospira* DNA in various biological samples, such as blood and urine, potentially enabling earlier diagnosis than MAT [20,21]. This technique amplifies specific regions of the *Leptospira* genome, allowing for the detection of even minute quantities of bacterial DNA, which is particularly useful in early stages of infection when bacterial loads may be low. Because PCR directly detects the pathogen’s genetic material rather than relying on the host’s antibody response, it can provide a faster diagnosis, often within 24–48 h, compared to the delayed results of MAT [22,23,24]. 

Despite the increasing use of PCR for leptospirosis diagnosis, the relative performance of MAT and PCR in different epidemiological settings and with diverse *Leptospira* serovars needs further elucidation. This systematic review aims to synthesize the available evidence and provide much-needed clarity on the roles of MAT and PCR in the diagnosis of human leptospirosis.

Specifically, our objectives are as follows: (1) to identify the most suitable biological fluid for sampling (blood, urine, cerebrospinal fluid) in different stages of the disease; (2) to evaluate the diagnostic accuracy of PCR in blood and urine; (3) to assess the geographical implications of leptospirosis on diagnostic test selection; and (4) to propose evidence-based diagnostic algorithms to guide clinicians in selecting the most appropriate test based on the clinical presentation and disease stage.

## 2. Materials and Methods

### 2.1. Search Strategy

This study followed the Preferred Reporting Items for Systematic reviews and Meta-Analysis (PRISMA) guidelines [25]. A comprehensive literature search was conducted using PubMed, Google Scholar, and Semantic Scholar databases. The following search terms were used individually and/or in combination: “leptospirosis”, “Leptospira”, “leptospirosis testing”, “PCR”, “MAT”, and “leptospirosis diagnosis”. No time restrictions were applied. The search was limited to original, peer-reviewed articles published in English and employing the following study designs: controlled trials, observational cohort studies (retro- and prospective), cross-sectional studies, and case-control studies.

### 2.2. Study Selection Process

**Figure 1:**
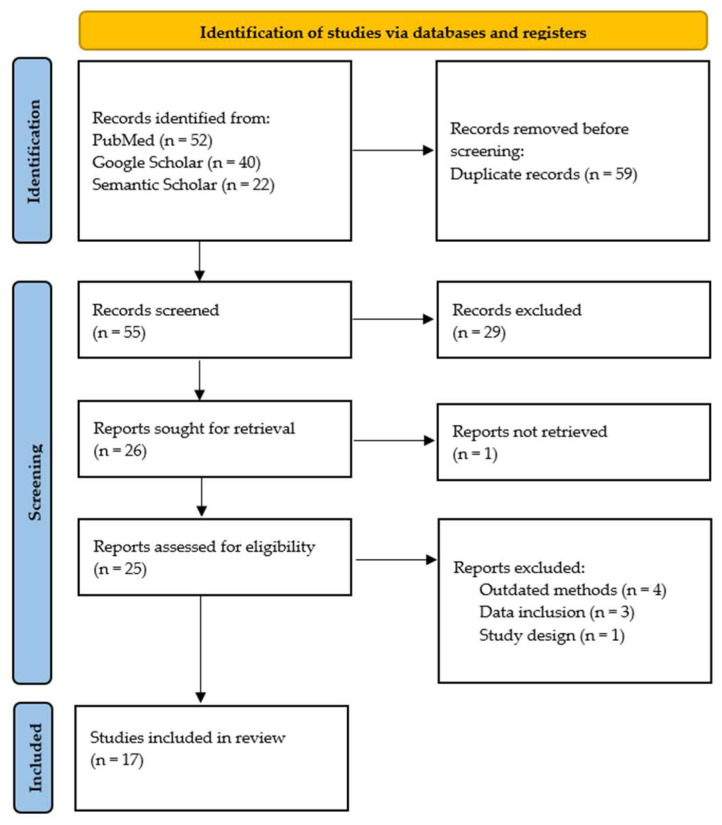
PRISMA flowchart of the study selection process.

Studies were included if they met the following criteria: (1) evaluated the diagnostic accuracy of PCR for human leptospirosis; (2) employed a case-control, controlled randomized trial, or cross-sectional study design; (3) included adult participants (age ≥ 18 years); and (4) were published in English. The studies were excluded for the following reasons: (1) they lacked data on PCR sensitivity and specificity; (2) they did not report on the specific PCR method used; (3) they were not published in peer-reviewed journals; (4) they did not have an English translation available; (5) they were not performed on humans; or (6) they employed a systematic review or meta-analysis methodology. To ensure reliability, two independent reviewers (C.P.B. and E.C.) screened all records. The inter-rater reliability was assessed using Cohen’s kappa, resulting in a value of 0.83, indicating very high agreement. Discrepancies were resolved through consensus or by a third reviewer (N.I.V.). The study selection process followed PRISMA guidelines and is illustrated in Figure 1.

This review excluded studies lacking sufficient participants to assess PCR diagnostic accuracy in human leptospirosis, omitting key accuracy measures (e.g., sensitivity, specificity), unspecified PCR techniques, or non-peer-reviewed sources to ensure data reliability. Research on non-human subjects, systematic reviews, and meta-analyses were also excluded to focus on original human studies and avoid result duplication.

### 2.3. Data Extraction

Following the study selection, two evaluators (E.C. and D.M.) independently reviewed all included publications. The data extraction was performed using a standardized form. The extracted information included the following: authors’ names, year of publication, study design, country where the study was performed, number of participants, leptospirosis testing method, PCR sensitivity and specificity, and biological samples tested. The discrepancies were resolved through consensus with a third reviewer (C.P.B.).

### 2.4. Risk of Bias Assessment

The risk of bias in included studies was assessed using the Quality in Prognosis Studies (QUIPS) tool, as described by Hayden et al. [26]. QUIPS evaluates six domains to determine the potential for bias: study participation, prognostic factor measurement, outcome measurement, study confounding, statistical analysis and reporting, and study funding. Each domain was categorized as low, moderate, or high risk of bias based on QUIPS criteria. Two assessors independently performed the risk of bias assessment, and discrepancies were resolved by consensus or in consultation with a third reviewer (N.I.V.). A breakdown of the QUIPS domains and the specific ratings, tailored specifically for this study, can be found in Table S1 of the Supplemental Material.

## 3. Results

### 3.1. Overview of Included Studies

Following the initial search in the databases (PubMed, Semantic Scholar, and Google Scholar) using the aforementioned keywords, 114 articles were identified. After removing 59 duplicates, 55 articles remained for the screening process. Applying the predefined exclusion criteria led to the exclusion of 30 articles (studies not published in English or no English translation available, studies not published in peer-reviewed journals, etc.). Further screening for eligibility through full-text analysis led to the exclusion of eight additional studies due to various reasons, including inappropriate study design, lack of relevant data, focus on unrelated topics, etc. Figure 1 describes the study selection process. Ultimately, 17 articles were included in this systematic review [20,23,27,28,29,30,31,32,33,34,35,36,37,38,39,40,41]. These studies reported on contemporary and effective methods for the laboratory diagnosis of leptospirosis. Table 1 provides an overview of these studies, including study design, geographical location, sample size, sensitivity and specificity of PCR testing, additional laboratory tests employed, and the biological sample used for testing. Table S2 of the Supplemental Material provides a list of studies excluded after full-text analysis, along with the reason for exclusion [22,42,43,44,45,46,47,48]. 

### 3.2. Risk of Bias Assessment

The assessment of risk of bias across the 17 studies revealed that the QUIPS domains with the highest risk were “Outcome Measurement” and “Study Confounding”. Six studies (35.3%) were rated as “high risk” in these domains due to limitations in describing statistical methodologies, patient groups, and data utilization, as well as the selective reporting of statistically significant results. The specific risk of bias ratings for each included study is detailed in Table S3 of the Supplemental Material.

### 3.3. The Role and Clinical Application of PCR Testing in Leptospirosis

Our review of 17 studies highlights the crucial role of PCR in diagnosing human leptospirosis, particularly during the acute phase of the disease. Overall, the evidence strongly supports the use of PCR as a valuable tool for diagnosing leptospirosis, particularly in the early stages. Its rapid turnaround time, high sensitivity, and ability to detect *Leptospira* DNA before antibodies develop make it a crucial addition to the diagnostic arsenal for this potentially life-threatening disease.

#### 3.3.1. PCR Performance in Early Diagnosis

Several studies emphasized the superior performance of PCR in the early stages of leptospirosis. Fonseca et al. [27] analyzed 124 serum samples and found that PCR had a sensitivity of 62% in the first 3–8 days of illness, higher than MAT (69%), IgM ELISA (79.3%), and SAT (72.4%). However, they noted that PCR sensitivity decreased in subsequent samples (72.7% in days 9–14 and 44.4% in days 15–42), while the sensitivity of serological tests increased. Perwez et al. [29] analyzed 100 blood samples and found that PCR had a sensitivity of 80% and specificity of 90%. They emphasized that PCR is particularly valuable for detecting *Leptospira* DNA in the very early stage of the disease, even before the appearance of IgM antibodies. Riediger et al. [31] also observed higher sensitivity (86%) for PCR in samples collected within the first six days of disease onset, compared to 34% sensitivity in samples collected after 7 days. Ahmed et al. [34] developed a multiplex PCR assay that demonstrated high sensitivity for detecting *Leptospira* DNA, even at low concentrations (1 × 10^3^ leptospires/mL). This high sensitivity makes it a promising tool for early diagnosis, potentially enabling rapid identification and treatment. Agampodi et al. [32] found that PCR had the highest sensitivity (74%) among all tests used (MAT, ELISA, and qPCR) during the acute phase of leptospirosis. This highlights the value of PCR in early detection when other diagnostic methods may have lower sensitivity.

These findings consistently demonstrate the value of PCR in rapidly identifying leptospirosis during the acute phase, when prompt treatment is crucial.

#### 3.3.2. Comparison with Other Diagnostic Tests

Many studies directly compared PCR with other diagnostic methods, often showing its superiority in early detection. Agampodi et al. [32] found that PCR was the most sensitive test during the acute phase of leptospirosis, detecting 74% of confirmed cases. In contrast, IgG ELISA detected only 35.5%, and MAT detected 12% of cases during this early stage. Furthermore, they found that 10 out of 40 patients with paired sera were PCR positive, while 5 of those were negative by paired-sample MAT, highlighting the ability of PCR to detect cases missed by MAT. Interestingly, only five MAT-positive samples were detected using PCR, underscoring the complementary nature of these tests and the potential value of a combined approach. 

Perwez et al. [29] conducted a study on 100 blood samples, comparing PCR and ELISA for leptospirosis diagnosis. PCR detected *Leptospira* DNA in 34 samples, while ELISA identified IgM antibodies in 35 samples. Notably, 28 samples were positive by both methods. PCR demonstrated a sensitivity of 80% and specificity of 90%, highlighting its effectiveness as a sensitive and specific diagnostic tool, especially in the early stages of infection. Mullan et al. [28] showed that PCR had a moderate sensitivity of 52% but a high specificity of 79% compared to the gold standard MAT test. While the sensitivity was lower than some other studies, the high specificity indicates that PCR can be a useful tool for ruling in leptospirosis, especially when combined with other diagnostic tests or in cases with a high clinical suspicion. 

Smythe et al. [38] reported that PCR had a higher detection rate than MAT in clinical specimens, particularly in urine samples. They found that PCR could detect leptospiral DNA in urine samples with a detection limit of approximately 10 cells. Moreover, their PCR assay demonstrated high specificity, with detectable amplification products exclusively for pathogenic strains of *Leptospira*. The non-pathogenic strains and other bacterial species did not produce amplification products, confirming the specificity of the test. Wangroongsarb et al. found that PCR had a sensitivity of 80% and specificity of 96.2% when compared to culture/MAT, demonstrating its effectiveness as a diagnostic tool. They also highlighted the advantages of PCR in terms of faster turnaround time, taking only a few hours compared to the longer processing time required for culture. 

Philip et al. [23], Blanco et al. [40], and Wangroongsarb et al. [41] also emphasized the advantage of PCR in early diagnosis, as it can detect leptospiral DNA before antibodies are detectable by serological methods. This is particularly important for patients presenting with acute symptoms who may not yet have developed a detectable antibody response, allowing for earlier intervention and potentially preventing severe complications.

These findings suggest that PCR can be a valuable addition to, or even replacement for, traditional serological tests, particularly in the early stages of leptospirosis. See Table 2 for a summary of PCR comparisons with other diagnostic methods. 

#### 3.3.3. Combined Testing Strategy 

Several studies emphasized the importance of combining PCR with serological tests like MAT to maximize diagnostic accuracy, particularly given the varying sensitivities of each method at different stages of illness. Fonseca et al. [27] found that while PCR was more sensitive than serological tests (MAT, IgM ELISA, and SAT) in initial serum samples collected within 3–8 days of illness, its sensitivity decreased in later samples. Conversely, the sensitivity of serological tests increased over time. They concluded that combining PCR with serological tests improved the sensitivity of diagnosis in the early phase of the disease, capturing cases that might be missed by a single test alone. 

Agampodi et al. [35] demonstrated that a combined testing strategy using both PCR and MAT was necessary for maximal sensitivity. They found that 10 out of 40 patients with paired sera were PCR positive in the acute phase, while 5 of those were negative by paired-sample MAT. Conversely, only five MAT-positive samples were detected using PCR. This highlights the complementary nature of these tests, with PCR excelling in early detection and MAT becoming more reliable as antibodies develop.

Waggoner et al. [36] strongly advocated for a combined testing strategy for acute leptospirosis, including both molecular and serologic testing. They found that adding PCR to MAT for all patients in their study increased the number of detected leptospirosis cases by 30.4%, from 102 (12.5%) to 133 (16.3%). This significant increase underscores the potential of combined testing to maximize case detection and improve diagnostic accuracy.

These findings consistently highlight the value of a combined testing approach, leveraging the strengths of both PCR and MAT to optimize leptospirosis diagnosis across different stages of the disease.

### 3.4. Comparative Utility of Blood and Urine Samples for PCR Testing

This review also aimed to identify the optimal biological fluid for PCR testing in human leptospirosis diagnosis. While various biological fluids can be used (blood, serum, urine, CSF, etc.), the included studies primarily focused on blood and urine. Each sample type has its advantages and disadvantages, and they can be used synergistically depending on the stage of illness and clinical context.

Blood and serum are particularly useful in the early phase of infection (first 7–10 days) when *Leptospira* circulates in the bloodstream. This makes blood PCR ideal for early detection before antibody development. Urine sampling becomes more relevant in later stages of infection as *Leptospira* localizes to the kidneys and is excreted in the urine. This makes urine PCR useful for detecting chronic or persistent infections. However, careful handling is required to avoid contamination, and urine PCR may yield false negatives in the early stages of the disease. While most studies used either blood or urine for PCR testing, some emphasized the potential value of combining both sample types for increased diagnostic accuracy across different stages of illness. 

Smythe et al. [38] compared the detection of *Leptospira* in blood and urine using PCR. The study found that both blood and urine can be valuable samples for PCR testing, with the real-time PCR assay detecting as few as 2 leptospiral cells in serum and 10 cells in urine. Blood may be more suitable in the early stages (acute phase up to 10 days) when bacteria circulate in the bloodstream, while urine may be more relevant in later stages when leptospires are excreted in the urine. The authors acknowledge that the lower sensitivity in urine (detection limit of 10 cells vs. 2 cells in serum) is unclear and requires further investigation, possibly due to PCR-inhibiting components in urine or less efficient DNA extraction. They emphasize the need for further research to optimize PCR testing in urine and address potential challenges.

### 3.5. Descriptive Analysis of the Sensitivity and Specificity of PCR Testing 

Given the heterogeneity of the included studies with respect to design, population, and methodology, a descriptive statistical analysis was considered appropriate. Analysis of the objective indices (sensitivity and specificity of PCR in human leptospirosis) across 11 specialized studies—selected because they reported either sensitivity or specificity, or both—revealed an average PCR sensitivity of 75%, with a range of 51% to 100%. The average PCR specificity was 93.85%, ranging from 79% to 100%. While specificity demonstrated consistently high values, sensitivity exhibited greater variability among the included studies. Nonetheless, with a median sensitivity of 74.2% and an average of 75%, PCR appears to be a sensitive method for the early diagnosis of human leptospirosis. These findings are summarized in Table 3.

### 3.6. PCR Techniques in Leptospirosis: Methods and Key Markers

**Figure 2:**
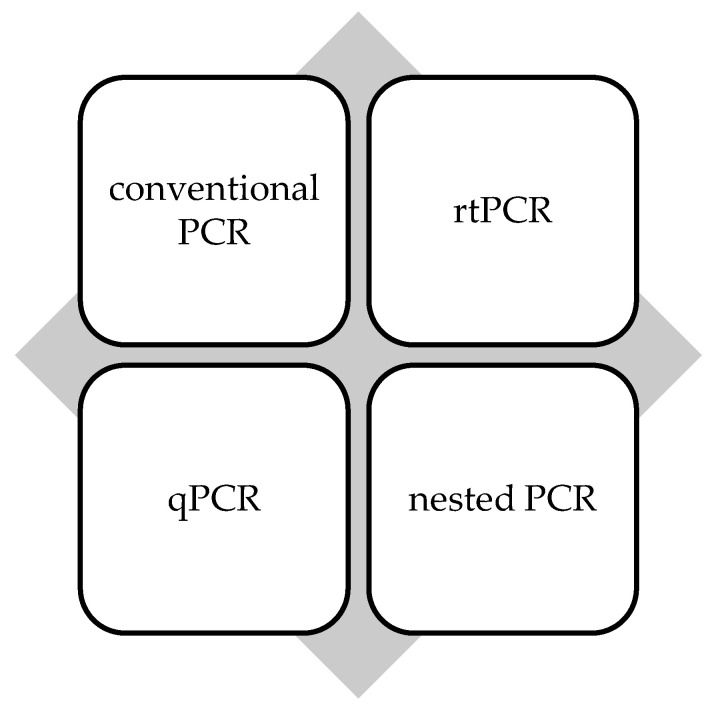
PCR methods used in the included studies.

In the studies included in the review, various types of PCR techniques were used for the diagnosis of leptospirosis, and the molecular markers analyzed varied depending on the specific study. In general, the use of different types of PCR, including conventional PCR, real-time PCR (rtPCR), multiplex PCR, and nested PCR (Figure 2), each with advantages and limitations in the diagnosis of leptospirosis, is observed. The most commonly used techniques and markers are detailed below.

Conventional PCR was used in the following studies: Fonseca et al. [27], Mullan et al. [28], and Perwez et al. [29]. This type of conventional PCR has the advantage of affordability but may be less sensitive than real-time PCR or methods.

Real-time PCR (rtPCR) was used in the studies of Levett et al. [20], Ahmed et al. [34], and Waggoner et al. [36]. This technique allows real-time quantification of PCR products and can provide higher sensitivity and specificity. An example is the use of rtPCR targeting lipL32. 

Quantitative PCR (qPCR) was used in Agampodi et al. [32], Sreevalsan et al. [33], and Agampodi et al. [35] as an advanced PCR method that allows the measurement of the amount of *Leptospira*-specific DNA in a sample. It is usually used to assess genes such as 16S rRNA and lipL32. 

Nested PCR was used in the studies of Blanco et al. [40] and Philip et al. [23]. This being a two-step method, which increases the sensitivity of the assay, is useful in cases with small amounts of genetic material.

**Figure 3:**
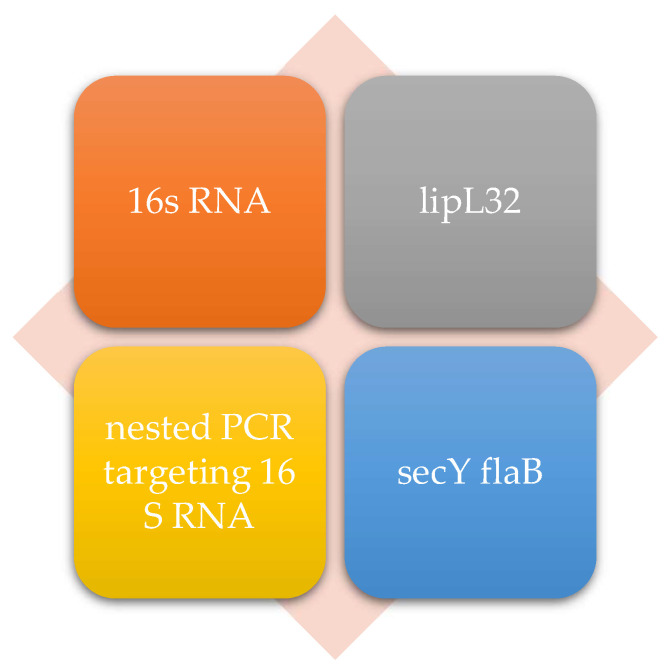
Main molecular markers in the diagnosis of leptospirosis.

In terms of target genes and proteins, 16S rRNA genes were commonly used in the studies of Agampodi et al. [32], Wangroongsarb et al. [41], and Philip et al. [23]. This is a standardized marker used for the detection of *Leptospira*, having the advantage of sensitivity and the ability to identify multiple *Leptospira* species (Figure 3). Another essential marker used in diagnostics is the LipL32 protein, used in real-time PCR, such as in the studies of Riediger et al. [31], Waggoner et al. [36], and Levett et al. [20]. This is a specific antigen for pathogenic *Leptospira* and is often used due to its high specificity. Nested PCR targeting 16S rDNA served as a method in Philip et al. [23] and Blanco et al. [40]. Nested PCR is used to amplify conserved regions of 16S rDNA, thus improving the sensitivity of the assay, especially in cases of infections with low bacterial loads. From a strictly theoretical point of view, the SecY and flaB genes are also analyzed in some studies to increase diagnostic accuracy. They are involved in bacterial flagella formation and are considered conserved in *Leptospira* species.

### 3.7. The Impact of Geographical Region on Test Selection for Leptospirosis Diagnosis

The data from Table 1 illustrate how the sensitivity and specificity of PCR assays vary across clinical studies conducted in different geographical regions, reflecting differences in diagnostic test selection, technological capabilities, and regional healthcare infrastructure. In resource-limited settings, such as Sri Lanka and India, studies predominantly employed conventional PCR or quantitative PCR (qPCR) methods, which are more accessible and cost-effective but may lack the sensitivity of advanced assays. In contrast, regions with more developed healthcare systems, such as The Netherlands and Brazil, frequently utilized sophisticated PCR techniques, including real-time PCR (rtPCR) or qPCR targeting the 16S rRNA gene, which offer higher sensitivity and specificity. This disparity underscores how diagnostic choices are influenced not only by technological availability but also by economic constraints and laboratory expertise. The type of biological sample used for PCR testing also varies geographically, likely due to differences in infrastructure and clinical practices. Studies from resource-limited regions often relied on blood samples, which are easier to collect and process in settings with basic laboratory facilities (e.g., Perwez et al. [29]), whereas studies in regions with advanced infrastructure, such as Smythe et al. [38], incorporated both blood and urine, leveraging greater capacity for sample handling and analysis. This variation may reflect logistical factors, such as the availability of refrigeration for urine storage or the ability to perform rapid DNA extraction.

## 4. Discussion

Our systematic review highlights the significant potential of polymerase chain reaction (PCR) in diagnosing human leptospirosis, particularly during the early stages of infection. While the Microscopic Agglutination Test (MAT) remains the traditional gold standard, PCR offers several advantages, including rapid turnaround time and the ability to detect *Leptospira* DNA before antibodies become detectable. This is crucial for prompt treatment initiation and improved patient outcomes, especially in severe cases.

The superior performance of PCR in the acute phase of leptospirosis is attributed to its ability to directly detect the pathogen’s genetic material when bacterial loads are highest in the bloodstream [27,29,30,31]. This allows for rapid identification of the pathogen, often before antibodies are detectable by serological methods. However, as the disease progresses, bacterial DNA may become less abundant in blood as *Leptospira* disseminates to other organs and is cleared by the immune system or antibiotic treatment. This can lead to a decline in PCR sensitivity over time, highlighting the importance of prompt sample collection and testing. The rapid turnaround time of PCR facilitates prompt treatment initiation, which is crucial in leptospirosis to prevent severe complications such as Weil’s disease or pulmonary hemorrhage syndrome. This highlights the importance of timely sample collection and prompt testing to maximize the diagnostic accuracy of PCR. 

While PCR demonstrates excellent performance in early diagnosis, our review emphasizes the value of a combined testing strategy incorporating both molecular and serological methods. PCR excels in early detection when antibody levels may be low, while serological tests like MAT become more reliable as antibodies develop later in the disease course. This complementary approach maximizes diagnostic accuracy and ensures that cases are not missed, regardless of the stage of illness [27,32,35,36].

Our review also examined the comparative utility of blood and urine samples for PCR testing. Blood and serum are ideal for early detection when *Leptospira* circulates in the bloodstream, while urine may be more suitable in later stages or for detecting chronic infections as the bacteria are shed in the urine. The choice between blood and urine for PCR testing should be guided by the stage of illness, clinical suspicion, and the need for rapid diagnosis.

Our study also highlights the potential limitations of the PCR method in leptospirosis. Several factors can influence its performance and potentially lead to inaccurate results. For instance, prior antibiotic treatment can reduce bacterial load, making it difficult for PCR to detect *Leptospira* DNA, even if the patient is infected. For example, Fonseca et al. [27] reported that PCR sensitivity decreased in subsequent samples collected after the first week, potentially due to antibiotic treatment affecting bacterial load. Similarly, Riediger et al. [31] observed a decrease in PCR sensitivity over time, with higher sensitivity in samples collected withing the first 6 days of disease onset, compared to those collected after 7 days. This observation could also be attributed to the effect of antibiotic treatment on bacterial load. Genetic variations in the target regions of the bacterial genome can affect the binding of PCR primers, potentially leading to false negatives [20,34]. Furthermore, the high sensitivity of PCR makes it susceptible to contamination, especially from reagents used in the process. This can lead to false-positive results, highlighting the need for meticulous laboratory practices and stringent quality control measures [20,38]. Additionally, certain substances in clinical samples, such as heparin, a commonly used anticoagulant, can inhibit the enzymes involved in PCR, resulting in false-negative results [49,50].

Our review underscores the importance of employing a combined testing strategy that leverages the strengths of both PCR and MAT. While PCR excels in early detection, MAT provides valuable confirmation and serological information later in the disease course. This complementary approach maximizes diagnostic accuracy and ensures that cases are not missed, regardless of the stage of illness [7,32,35,36,38]. By combining the rapid detection capabilities of PCR with the confirmatory power of MAT, clinicians can optimize patient management and ensure timely intervention.

Our findings have important implications for clinical practice. In suspected cases of leptospirosis, particularly those presenting early in the disease course, PCR should be considered a first-line diagnostic test, especially when rapid diagnosis is crucial. Given that *Leptospira* initially circulates in the bloodstream, blood PCR is preferred for early diagnosis (within the first week of illness). As the infection progresses, *Leptospira* colonizes the kidneys and then is eliminated through urine. Therefore, urine may be the preferred sample for PCR testing after the first week of illness, as its sensitivity increases with higher bacterial load in the urine. However, in developing countries, the availability of PCR may be limited by inadequate laboratory infrastructure and high costs, necessitating reliance on simpler serological tests like MAT in resource-constrained settings. Strengthening diagnostic capacity through affordable, portable PCR technologies could bridge this gap and enhance early detection in such regions. 

This systematic review has some limitations. The heterogeneity of the included studies, in terms of design, population, and methodology, limited the ability to conduct a meta-analysis. This heterogeneity may have contributed to the variability observed in PCR sensitivity across studies. Future research could focus on standardizing PCR protocols and conducting larger, multi-center studies to generate more robust estimates of sensitivity and specificity. Additionally, the review primarily focused on studies published in English, potentially introducing a language bias. Future reviews could incorporate a wider range of languages to ensure a more comprehensive assessment of the literature. Furthermore, while this review highlighted the value of PCR in diagnosing leptospirosis, the included studies primarily focused on its diagnostic accuracy. Future research could explore other aspects of PCR testing, such as its cost-effectiveness, feasibility in different healthcare settings, and impact on patient management and clinical outcomes. Investigating the potential of novel PCR techniques, such as multiplex PCR or isothermal amplification methods, could further enhance the diagnostic capabilities for leptospirosis. Finally, exploring the role of PCR in differentiating between various *Leptospira* species and serotypes could have significant implications for epidemiological surveillance and targeted treatment strategies.

## 5. Conclusions

This systematic review of 17 studies provides compelling evidence for the value of PCR in diagnosing human leptospirosis. PCR demonstrates high sensitivity and specificity, particularly in the early stages of the disease, making it a valuable tool for rapid and accurate diagnosis. Our analysis revealed an average PCR sensitivity of 75% and specificity of 93.85% across 22 specialized studies. While PCR sensitivity can vary depending on several factors, its ability to detect *Leptospira* DNA before antibodies develop makes it a crucial addition to the diagnostic arsenal. Furthermore, combining PCR with serological tests like MAT can maximize diagnostic accuracy across different stages of illness.

## Figures and Tables

**Table 1:** Analysis of PCR diagnostic sensitivity, specificity, and laboratory testing parameters in clinical studies.

Study ID	Study Design	Region	Participants	Diagnosis Test	Sensitivity of PCR	Specificity of PCR	Laboratory Tests	Biological Samples	PCR Technique Used	Target Gene/Protein
Fonseca 2006 [27]	case-control	Brazil	124	PCR	62%	100%	MAT, IgM ELISA, SAT, PCR	Blood	Conventional PCR	lipL32
Mullan 2016 [28]	prospective observational	South Gujarat, India	207	PCR	52%	79%	Rapid leptocheck test, IgM ELISA, MAT, *Leptospira* culture, PCR	Blood	Conventional PCR	16S rRNA
Perwez 2011 [29]	cross-sectional observational	India	100	PCR	80%	90%	IgM ELISA, PCR,	Blood	Conventional PCR	flaB
Shekatkar 2010 [30]	observational	India	100	-	-	-	MAT, IgM ELISA, PCR	Blood	PCR	16S rRNA
Riediger 2017 [31]	prospective case-control	Brazil	150	PCR	60.60%	98.00%	PCR targeting lipL32	Blood	PCR targeting lipL32	lipL32
Agampodi 2012 [32]	observational	Sri Lanka	381	PCR	51.00%	-	qPCR targeting 16s RNA gene	Blood	qPCR	16S rRNA
Sreevalsan 2024 [33]	multi-site observational	India	67	PCR	90%	-	IgM ELISA, PCR,	Blood	Conventional PCR	lipL32
Ahmed 2009 [34]	prospective observational	Netherlands	133	rtPCR	100%	93-100%	PCR	Blood, urine	rtPCR	lipL32
Levett 2005 [20]	retrospective observational	USA	-	rtPCR	-	-	real-time PCR targeting lipL32	Blood, Urine	rtPCR	lipL32
Agampodi 2016 [35]	observational	Sri Lanka	96	qPCR	74.20%	-	qPCR, two ELISA, MAT	Blood	qPCR	16S rRNA
Waggoner 2015 [36]	placebo-controlled trial	Brazil	818	PCR	81%	99%	LEPTO-MD, rtPCR	Blood	LEPTO-MD, rtPCR	lipL32
Katz 2012 [37]	case-control study prospective cohort study	-	-	qPCR	-	-	qPCR, MAT	Blood	qPCR	16S rRNA
Smythe 2002 [38]	prospective observational	Australia	-	PCR	-	-	qPCR	Blood, Urine	qPCR	16S rRNA
Bourhy 2011 [39]	cross-sectional observational	France	-	qPCR	-	-	qPCR	Blood	qPCR	16S rRNA
Blanco 2014 [40]	retrospective observational	Brazil	521	Nested PCR	95.70%	-	MAT, nested PCR	Blood	Nested PCR	16S rDNA
Philip 2020 [23]	retrospective observational	Central Malaysia	165	PCR	-	-	MAT, qPCR targeting the lipL32, nested PCR targeting 16S rDNA	Blood	qPCR, Nested PCR	lipL32, 16S rDNA
Wangroongsarb 2005 [41]	prospective observational	Thaliand	93	PCR	80%	96.20%	PCR targeting 16SrRNA, MAT, culture	Blood	PCR targeting 16S rRNA	16S rRNA

**Table 2:** Comparison of PCR with other diagnostic tests for leptospirosis.

Study ID	Diagnostic Methods Compared	PCR Sensitivity (%)	PCR Specificity (%)	Key Findings and Observations
Agampodi et al. [32]	PCR, IgG ELISA, MAT	74	-	PCR most sensitive in acute phase (74%), vs. IgG ELISA (35.5%) and MAT (12%). Detected cases missed by MAT (10/40 PCR+ vs. 5/40 MAT+). Suggests complementary use.
Perwez et al. [29]	PCR, IgM ELISA	80	90	PCR detected *Leptospira* DNA in 34/100 samples, IgM ELISA in 35/100 (28 overlap). Effective in early stages before IgM detectable.
Mullan et al. [28]	PCR, MAT, Rapid Leptocheck, IgM ELISA	52	79	Moderate sensitivity but high specificity vs. MAT. Useful for ruling in leptospirosis when combined with other tests.
Smythe et al. [38]	PCR, MAT	-	High (pathogenic-specific)	Higher detection rate in urine vs. MAT. Specific to pathogenic Leptospira (no amplification in non-pathogenic strains).
Wangroongsarb et al. [41]	PCR, MAT, Culture	80	96.2	Faster turnaround (hours) vs. culture/MAT. Effective early diagnostic tool.
Philip et al. [23]	PCR, MAT	-	-	PCR advantageous for early diagnosis before serological response detectable.
Blanco et al. [40]	Nested PCR, MAT	95.7	-	Emphasized PCR’s early detection capability vs. MAT, especially pre-antibody development.

**Table 3:** Sensitivity and specificity of PCR in human leptospirosis.

Descriptive Statistics	Sensitivity of PCR	Specificity of PCR
MAX	100%	100%
MIN	51%	79%
MEDIAN	74.2%	96.2%
AVERAGE	75%	93.85%
ST DEV	0.200	0.071

## Data Availability

The original contributions presented in the study are included in the article. Further inquiries can be directed to the corresponding author.

## Supplementary Materials

The following supporting information can be downloaded at: https://www.mdpi.com/article/10.3390/microorganisms13030667/s1, Table S1: QUIPS Domains for Risk of Bias Assessment; Table S2: Excluded Studies and Reason for Exclusion; Table S3: Risk of Bias Ratings.
