# Supplementary material for: Diagnostic Advances in Leptospirosis: A Comparative Analysis of Paraclinical Tests with a Focus on PCR
Source: Microorganisms. 2025 Mar 15;13(3):667. doi: 10.3390/microorganisms13030667 (PMC11944366; doi:10.3390/microorganisms13030667)
Supplement: Supplementary file 1 [file microorganisms-13-00667-s001.zip › microorganisms-3401251-supplementary.pdf]

Systematic Review

Diagnostic Advances in Leptospirosis: A Comparative Analysis of Paraclinical Tests with a Focus on PCR

Supplemental Material

Table S1. QUIPS Domains for Risk of Bias Assessment**.**

Domain	Criteria	Risk of bias rating
1. Study enrolment	The study population and key characteristics are thoroughly described. Eligibility criteria and recruitment processes are transparently detailed. The study period and location are specified. Adequate participation of eligible individuals is ensured, with no exclusions based on biomarker values or patient outcomes.	a. Low Risk: All criteria are met, suggesting minimal differences between participants and eligible non-participants.<br>b. Moderate Risk: Some criteria are unclear or problematic.<br>c. High Risk: Multiple criteria are problematic, indicating potential differences between participants and eligible non-participants.
2. Study attrition	No unwarranted exclusions or significant non-participation are observed. Efforts to collect data from participants who dropped out are described (if applicable). No major differences exist between participants who completed the study and those who did not (in cases of justified exclusions).	a. Low Risk: All criteria are met, suggesting minimal differences between completing and non-completing participants.<br>b. Moderate Risk: Some criteria are unclear or problematic.<br>c. High Risk: Multiple criteria are problematic, indicating potential differences between completing and non-completing participants.
3. Biomarker measurement	The laboratory technique or method for measuring the biomarker is clearly reported. The same method and setting are used for all participants. Measurement methods are accurate, valid, consistent, and reliable. Continuous variables are handled appropriately, with rationale provided for any categorization. An adequate proportion of the study sample has complete data, or appropriate imputation methods are used.	a. Low Risk: All criteria are met, suggesting minimal impact on the relationship between the biomarker and outcome.<br>b. Moderate Risk: Some criteria are unclear or problematic.<br>c. High Risk: Multiple criteria are problematic, indicating potential impact on the relationship between the biomarker and outcome.
4. Outcome measurement	Mortality assessment methods are accurate, valid, consistent, and reliable. The method and setting of measurement are the same for all participants.	a. Low Risk: All criteria are met, suggesting minimal bias in outcome measurement.<br>b. Moderate Risk: Some criteria are unclear or problematic.<br>c. High Risk: Multiple criteria are problematic, indicating potential bias in outcome measurement.
5. Adjustment for other prognostic factors	All the important confounders (age, severity score) are measured and clearly defined. The method and setting of measurement are the	a. Low Risk: All criteria are met, suggesting minimal confounding effects.

	same for all participants. Validated scales are used for measurement. The two potential confounders are accounted for in the study design or analysis. Appropriate methods are used to handle missing data.	b. Moderate Risk: Some criteria are unclear or problematic.<br>c. High Risk: Multiple criteria are problematic, indicating potential confounding effects.
6. Statistical analysis and reporting	Sufficient data are presented to assess the adequacy of the analysis. The model-building strategy is acceptable and based on a conceptual framework. The analysis is appropriate for the study design. There is no selective reporting of results.	a. Low Risk: All criteria are met, suggesting minimal bias in analysis and reporting.<br>b. Moderate Risk: Some criteria are unclear or problematic.<br>c. High Risk: Multiple criteria are problematic, indicating potential bias in analysis and reporting.

Table S2. Excluded Studies and Reason for Exclusion.

Study ID	Title	Reason for exclusion
Yang 2019 [42]	Nucleic acid and antigen detection tests for leptospirosis.	INCOMPATIBLE STUDY DESIGN
Fraune 2013 [43]	Evaluation of the diagnostic value of serologic microagglutination testing and a polymerase chain reaction assay for diagnosis of acute leptospirosis in dogs in a referral center.	USE OF NON-HUMAN TISSUE OR BIOLOGIC FLUID
Martin 2022 [44]	The Evaluation of the Diagnostic Value of a PCR Assay When Compared to a Serologic Micro-Agglutination Test for Canine Leptospirosis	USE OF NON-HUMAN TISSUE OR BIOLOGIC FLUID
Esteves 2018 [45]	Diagnosis of Human Leptospirosis in a Clinical Setting: Real-Time PCR High Resolution Melting Analysis for Detection of Leptospira at the Onset of Disease	INCONCLUSIVE RESULTS
Bal 1994 [22]	Detection of leptospires in urine by PCR for early diagnosis of leptospirosis	OUTDATED METHODS
Brown 1995 [46]	Evaluation of the polymerase chain reaction for early diagnosis of leptospirosis.	OUTDATED METHODS
Merien 1995 [47]	Comparison of polymerase chain reaction with microagglutination test and culture for diagnosis of leptospirosis.	OUTDATED METHODS
Hernández-Rodríguez 2011 [48]	A comparison between polymerase chain reaction (PCR) and traditional techniques for the diagnosis of leptospirosis in bovines.	USE OF NON-HUMAN TISSUE OR BIOLOGIC FLUID

Table S3. Risk of Bias Ratings.

Study ID	D1-Study participation	D2-Study attrition	D3-Prognostic factor measurement	D4-Outcome measurement	D5-Study confounding	D6-Statistical analysis and reporting
Fonseca 2006 [27]	Low	Low	Low	Low	Low	Low
Mullan 2016 [28]	Low	Moderate	Low	Moderate	Low	Low
Perwez 2011 [29]	Low	Low	Low	Low	Low	Low

Shekatkar 2010 [30]	Low	Moderate	Low	Moderate	High	Moderate
Riediger 2017 [31]	Moderate	Low	Moderate	Low	Low	Low
Agampodi 2012 [32]	Low	Low	Low	Moderate	Low	Low
Sreevalsan 2024 [33]	Moderate	Low	Low	Moderate	Low	Low
Ahmed 2009 [34]	Moderate	Moderate	Low	Moderate	Moderate	Moderate
Levett 2005 [20]	Low	Low	Low	High	Low	Low
Agampodi 2016 [35]	High	High	High	Moderate	Moderate	Low
Waggoner 2015 [36]	Low	Moderate	Low	Low	Moderate	Low
Katz 2012 [37]	Low	Low	Low	Low	Low	Low
Smythe 2002 [38]	High	Moderate	Moderate	High	Low	High
Bourhy 2011 [39]	High	Moderate	Low	High	High	High
Blanco 2014 [40]	Moderate	Moderate	Moderate	Moderate	High	Low
Philip 2020 [23]	Low	Low	Moderate	Low	Moderate	Low
Wangroongsarb 2005 [41]	Moderate	Moderate	High	Moderate	Low	Moderate
